# Two doses of botulinum toxin type A for the treatment of trigeminal neuralgia: observation of therapeutic effect from a randomized, double-blind, placebo-controlled trial

**DOI:** 10.1186/1129-2377-15-65

**Published:** 2014-09-27

**Authors:** Haifeng Zhang, Yajun Lian, Yunqing Ma, Yuan Chen, Caihong He, Nanchang Xie, Chuanjie Wu

**Affiliations:** 1 Department of Neurology, the First Affiliated Hospital, Zhengzhou University, 1 Jianshe East R, Zhengzhou City, HeNan Province 450052, People’s Republic of China

**Keywords:** Botulinum toxin type A, Trigeminal neuralgia, Double-blind, Placebo-controlled treatment

## Abstract

**Background:**

In the majority of cases, trigeminal neuralgia (TN) is a unilateral condition with ultra-short stabbing pain located along one or more branches of the trigeminal nerve. Although prophylactic pharmacological treatment is first choise, considering of insufficient effect or unacceptable side effects, neurosurgical treatment or lesion treatment should be considered. In addition to all these procedures mentioned above, one approach has been based on local intradermal and/or submucosal injections of Botulinum Toxin Type A (BTX-A).

**Methods:**

We conducted a randomized, double-blind, placebo-controlled since November 2012, and adopted local multi-point injection in 84 cases of classical TN with different doses of BTX-A. Eighty four patients were randomized into following groups: placebo (n = 28); BTX-A 25U (n = 27); BTX-A 75U (n = 29). Follow-up visits were conducted every week after the injection, and the overall duration of the study for each patient were 8 weeks to observe the pain severity, efficacy and adverse reactions at endpoint.

**Results:**

The visual analogue scale (VAS) scores of 25U and 75U groups reduced significantly compared to placebo as early as week 1, and sustained until week 8 throughout the study. There was no significant difference in VAS between 25U and 75U groups throughout the study. The response rates of 25U group (70.4%) and 75U group (86.2%) were significantly higher than placebo group (32.1%) at week 8, and there was no significant difference between 25U and 75U groups. Evaluation of the Patient Global Impression of Change (PGIC) demonstrated that 66.7% (25U group) and 75.9% (75U group) of the patients reported that their pain symptoms were ‘much improved’ or ‘very much improved’ versus 32.1% of the placebo group, and there was also no significant difference between 25U and 75U groups. All adverse reactions were graded as mild or moderate.

**Conclusions:**

BTX-A injection in TN is safe and efficient. It is a useful treatment for refractory TN. Lower dose (25U) and high dose (75U) were similar in efficacy in short-term.

## Background

In the majority of cases, trigeminal neuralgia (TN) is a unilateral condition with ultra-short stabbing pain located along one or more branches of the trigeminal nerve. The pain is often triggered by stimuli such as chewing, washing of the face, speech and tooth-brushing [1]. It usually affects one side of the face, and occurs more often in the elderly and has a profound effect on quality of life. According to epidemiological studies, approximately 4–28.9/100,000 persons worldwide experience TN [2-4].

Trigeminal neuralgia is primarily caused by compression of the nerve near its origin at the pons. The treatment consists primarily of prophylactic pharmacological treatment with anti-epileptics. In case of insufficient effect or unacceptable side effects, neurosurgical treatment using microvascular decompression or lesion treatment should be considered [5]. In addition to all these procedures mentioned above, one approach has been based on local intradermal and/or submucosal injections of Botulinum Toxin Type A (BTX-A) [6].

BTX-A is one of the most potent neurotoxins from natural toxins and synthetic toxicant known, which blocks neuromuscular transmission through decreased acetylcholine release, resulting in muscle relaxation. It is also used in treating blepharospam and hemifacial spasm [7]. Additionally it is reported to be effective in the treatment of chronic migraine and may be a promising choice in headache treatment [8-11]. In some reports, BTX-A has been shown to be a new possible choice of treatment for TN, and may be an efficient, safe and novel strategy for TN treatment [6,12-19]. This therapy is simple and can be performed as an outpatient procedure without anesthesia, and the toxin does not cause skin sensory loss or dysesthesia. However, there is no large randomized controlled trial and relevant data except our study in 2012 [6], and the dose has not been standardised. The aim of the present study was to define an effective and safe dose of BTX-A for the treatment.

For this purpose, we conducted a randomized, double-blind, placebo-controlled since November 2012, and adopted local multi-point injection in 84 cases of classical TN with different doses of BTX-A. The primary objective was to further clarify the efficacy, safety and tolerability of intradermal and/or submucosal administration of different doses of BTX-A in patients with TN.

## Methods

### Study design

The study was a randomized, double-blind, placebo-controlled and parallel-group clinical study of BTX-A in the management of patients with TN. The overall duration of the study for each patient was 9 weeks, including a 1-week observation period to establish baseline pain symptoms, followed by a 8-week placebo run-in period. Follow-up visits were conducted every week after the injection.

The trial was approved by the local ethics committee of the First Affiliated Hospital of Zhengzhou University. The goal, procedure and possible adverse reactions of the study were explained to each patient before the study, and they were informed of the possibility of transient attendant risk of weakness and disfigurement produced by localized administration of BTX-A. Written informed consent was obtained from each patient. Patients were free to discontinue the trial at any time during the double-blind period.

The Patients were randomized into 3 groups: placebo groups received sterile isotonic saline injection and treatment group received 25U/1 mL (25U group) and 75U/1 mL (75U group) of BTX-A in the dermatome and/or mucosa (if the oral mucosa was involved) where pain was experienced. Patients were assigned to a computer-generated randomisation list, and randomization data were kept strictly confidential, accessible only by authorized persons. The data was locked and verified and unblended only when the trial was completed.

### Study participants

All included patients were recruited from the outpatients and inpatients who attended the neurology department of the First Affiliated Hospital of Zhengzhou University from Nov 2012 to Aug 2013. Each patient underwent MRI or CT to rule out the presence of structural pathology. All patients underwent detailed physical examination and data registration, including the history of present illness, past history, the auxiliary examination results, nervous system examination. Complete blood count (CBC), ECG, Liver function tests, renal function tests and other diagnostic tests should be done before the trial, to exclude coagulopathy, severe heart, liver, kidney and other organ dysfunction. According to the current version of the International Classification of Headache Disorders (ICHD-II) [20], and all patients were diagnosed with classical TN.

At baseline, patients usually received medications (e.g. carbamazepine, gabapentin, or opioids) to alleviate their pain. These medications remained unchanged during the course of the study. There were no new analgesic therapies at any time during the baseline or placebo run-in period.

Participants(>18 years), who were failure of recent treatment for TN at baseline (pain intensity mean score ≥ 4; mean attack frequency ≥ 4 per day; course > 4 months), were required to be in good general health, and understanding the possible complications such as transient facial weakness.

Exclusion criteria included: any disease that might put patients at increased risk if exposed to BTX-A (e.g. myasthenia gravis, motor neuron disease or Lambert-Eaton syndrome); an infection or skin problem at any of the injection sites; use of drug that damage the neuromuscular junction within 7 days before study entry (e.g. quinine, aminoglycosides or penicillamine); significant unstable medical disease or a history of a significant mental disorder; history of substance dependence or abuse. Furthermore, women who were pregnant, nursing, or planning a pregnancy during the study, or who were unable or unwilling to use a reliable form of contraception during the study were also excluded.

Patients who continued to meet all inclusion/exclusion criteria at the end of the baseline phase were randomized and proceeded to the double-blind period.

### Treatment

All of the treatment was administered in the treatment room at the Department of Neurology, the First Affiliated Hospital of Zhengzhou University, which is equipped with all the necessary facilities to ensure the safety of possible severe reactions or emergencies.

BTX-A (100U of Clostridium botulinum type A neurotoxin complex, 5 mg gelatin, 25 mg dextran, and 25 mg saccharose) was obtained from Lanzhou Biological Products Institute, China. Patients lay still in a supine position on a bed during the injections.

Patients were randomly assigned to receive treatment with placebo or total doses of 25Uor 75U of botulinum toxin type A (placebo group, 25U group, or 75U group) in a double blind prospective study design. The way to blind the study was to supply the study medication for each patient in three individual vials. Each vial contained either active botulinum toxin type A (25U or 75U) or matching placebo. All three vials were identical in appearance and were reconstituted with 1 ml saline solution (0.9%). For treatment 1 ml was drawn from vials, and the injections were administered intradermally and/or submucosally using a 1 mL syringe with a 0.45 × 16 mm needle.

The BTX-A or the same volume of isotonic saline were applied at 20 points, (0.05 ml) per point, between the epidermis and dermis of the skin where pain was experienced according to the patient’s description. The injections were conducted submucosally in the oral mucosa if the pain involved the oral mucosa. During the procedure, injection in deeper structures such as the muscles was avoided to prevent unwanted effects.

### Efficacy and safety measures

On awakening each morning, patients were required to provide a verifiable diary of their pain symptoms covering provoking factors, frequency of TN attacks, severity of pain (according to an 11-point visual analogue scale, VAS) and adverse reaction experienced during the previous 24 hours from baseline to endpoint. During the baseline phase, patient demographics, gender, and age were also recorded.

The following items were assessed during each follow up visit:

•Assessment of pain severity and pain attack frequency from baseline to endpoint.

•Global assessment of the overall response to treatment on the basis of the Patient Global Impression of Change (PGIC) scale. The PGIC is a self-evaluation of the patient’s overall change since the start of the study according to a seven-point scale (1, very much improved; 2, much improved; 3, minimally improved; 4, no change; 5, minimally worse; 6, much worse; 7, very much worse).

•The proportion of responders, defined as patient with ≥ 50% reduction in mean pain score from baseline to endpoint, was also used to assess the efficacy of the treatment.

•Safety was measured as the occurrence of adverse events and recorded with information including the date of onset, severity, duration, frequency, treatment required (if any), relationship to BTX-A treatment, and outcome.

### Statistical analysis

All analyses were performed on the intent-to-treat population and all statistical testing was two-sided. The quantitative data was assessed using the median value. Between groups comparisons were evaluated by means of the Kruskall-Wallis analysis to compare the age, duration of diseases, and pain intensity. Chi-square test was performed to assess the differences of the gender, PGIC distribution, and the proportion of responders among 3 groups. The SPSS 20.0 software package was used for statistical evaluation; p < 0.05 was considered statistically significant.

## Results

### Patient disposition

The recruitment period was between November 2012 and August 2013, with an 8-week follow-up period after the last patient was enrolled. A total of 95 patients were screened, and 84 were randomized into the following groups: placebo (n = 28); BTX-A 25U (n = 27); BTX-A 75U (n = 29). Four patients (one from 25U group, one from 75U group and two from placebo group) withdrew from the study owing to a lack of efficacy, leaving data on 80 patients for the final analysis. Overall, the groups were well matched for age, sex, duration of symptoms, pain severity, and pain attack frequency, and there were no significant differences at baseline (Table 1).

**Table 1:** Demographic characteristics and details at baseline

**Characteristics**		**Placebo (n = 27)**	**BTX-A (25u) (n = 25)**	**BTX-A (75u) (n = 28)**	**Total (n = 80)**	**P value**
Age, mean (SD), y	Mean (SD)	58.41 (11.74)	58.16 (11.54)	62.64 (13.32)	59.81 (12.30)	0.363
	Median	60	59	63	60	
	Min/max	31/78	41/80	40/89	31/89	
Mean months since onset of TN	Mean (SD)	50.96 (46.26)	91.96 (72.61)	72.64 (76.45)	71.36 (67.68)	0.181
	Median	36	96	36	48	
	Min/max	4/210	4/300	4/270	4/300	
Sex	Male	51.85%	40%	42.86%	45%	0.665
Pain intensity,VAS	Mean (SD)	6.96 (1.97)	6.24 (2.13)	7.18 (2.21)	6.81 (2.12)	0.197
	Median	7	5	6.5	6	
	Min/max	4/10	4/10	4/10	4/10	

### Pain severity-VAS score

**Figure 1:**
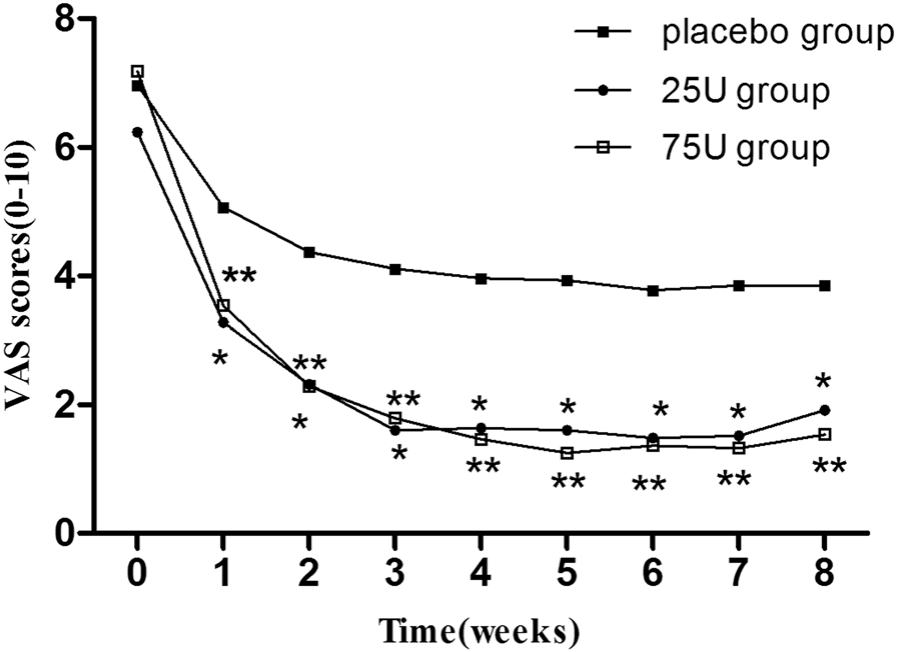
**Weekly mean scores as measured by VAS.** At baseline, each group was well matched for severity of their VAS scores. After injections, The VAS scores of 25U and 75U groups reduced significantly compared to placebo as early as week 1, and sustained until week 8 throughout the study (*P < 0.017, **P < 0.017) . There was no significant difference between 25U and 75U groups throughout the study (*P* > 0.05).

At baseline, each group was well matched for severity of their VAS scores (6.96 ± 1.97, 6.24 ± 2.13, and 7.18 ± 2.21 for placebo, 25U, and 75U groups respectively). After injections, The VAS scores of 25U and 75U groups reduced significantly compared to placebo as early as week 1, and sustained until week 8 throughout the study (*P < 0.017, **P < 0.017) (Figure 1). There was no significant difference between 25U and 75U groups throughout the study (*P* > 0.05) (Figure 1, Table 2).

**Table 2:** Compare 25U group and 75U group in VAS

**Week**	**Test statistic ︱R25 -‾R75︱**	**Standard error σR25 -‾R75**	**Standard test statistic Z**	**P**
1	−3.696	6.307	-.586	0.558
2	-.229	6.303	-.036	0.971
3	−1.258	6.255	-.201	0.841
4	2.709	6.208	0.436	0.663
5	5.166	6.205	0.833	0.405
6	2.099	6.200	0.339	0.735
7	2.588	6.211	0.417	0.677
8	3.784	6.230	0.607	0.544

Kruskall-Wallis test.

There was no significant difference between 25U and 75U groups throughout the study in the visual analogue scale (VAS) scores.

### Efficacy results

**Figure 2:**
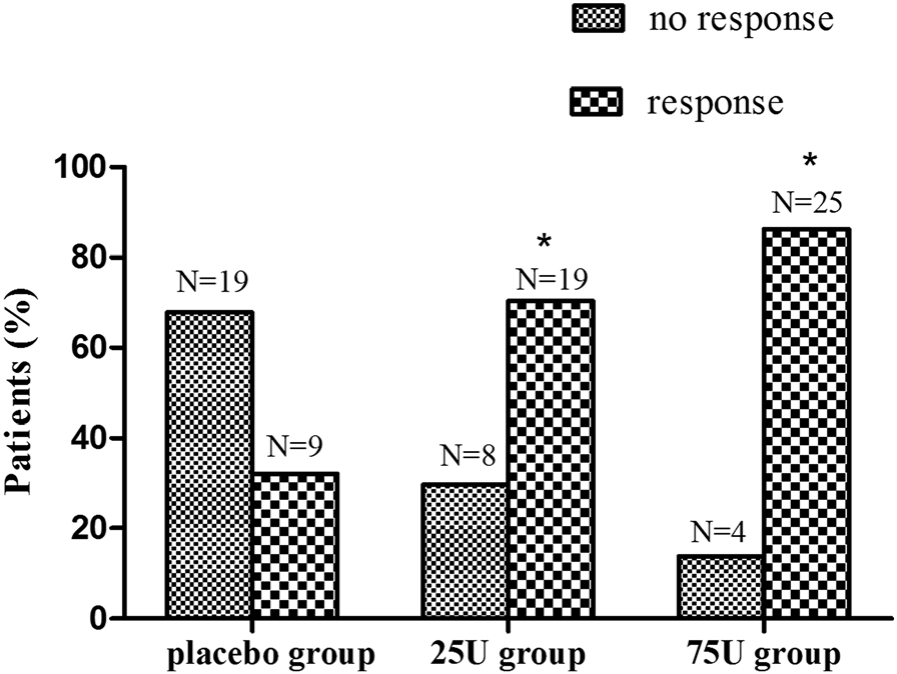
**The proportion of responders.** The response rates of 25U group (70.4%) and 75U group (86.2%) were significantly higher than placebo group (32.1%) at week 8 (*P < 0.017). There was no significant difference between 25U and 75U groups (*P* > 0.05).

The proportion of responders, defined as patient with ≥ 50% reduction in mean pain score from baseline to endpoint, was used to assess the efficacy. The response rates of 25U group (70.4%) and 75U group (86.2%) were significantly higher than placebo group (32.1%) at week 8(*P < 0.017). There was no significant difference between 25U and 75U groups (*P* > 0.05) (Figure 2).

**Figure 3:**
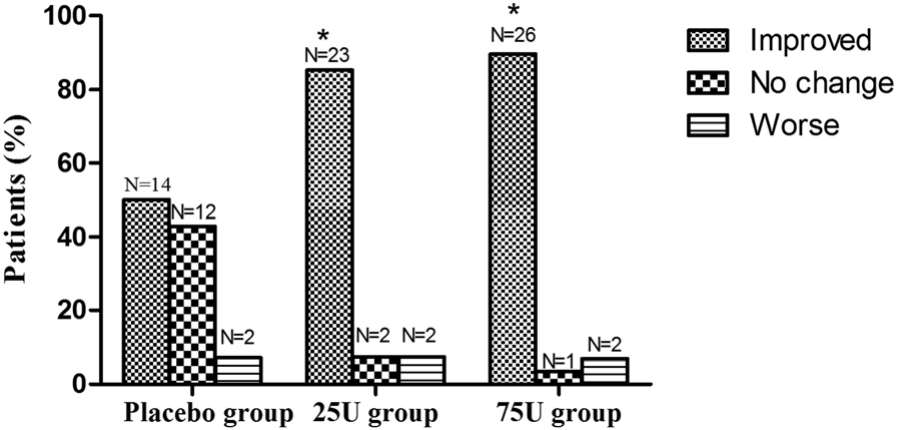
**Patient Global Impression of Change (PGIC) results.** Evaluation of the PGIC demonstrated that 66.7% of the patients in 25U group reported that their pain symptoms were ‘much improved’ or ‘very much improved’ versus 32.1% of the placebo group (*P < 0.017). In addition, there was a much higher proportion in 75U group (75.9%) compare to the placebo group (*P < 0.017), but there was no significant difference between 25U and 75U groups (*P* > 0.05).

Evaluation of the PGIC demonstrated that 66. 7% of the patients in 25U group reported that their pain symptoms were ‘much improved’ or ‘very much improved’ versus 32.1% of the placebo group (*P < 0.017). In addition, there was a much higher proportion in 75U group (*P < 0.017) (75.9%), but there was no significant difference between 25U and 75U groups (*P* > 0.05) (Figure 3).

### Safety and adverse reaction

Three patients (two in 25U group, one in 75U group) experienced short term facial asymmetry in the injection area during dynamic movement, which disappeared within 6 weeks. Transient edema in the injection area was observed in two patients (both in 25U group), and disappeared within 5 days. All adverse reactions were graded as mild or moderate, and none led to discontinuation of study.

## Discussion and conclusions

The International Association for the Study of Pain defines trigeminal neuralgia (TN) as “sudden, usually unilateral, severe, brief, stabbing, recurrent episodes of pain in the distribution of one or more branches of the trigeminal nerve”. Medical management is the first choice, including Carbamazepine, Oxcarbazepine, Bacloten, Lamotrigine, Gabapentin and ropivacaine. When drug treatments fail due to pharmacoresistance or intolerability of side effects, surgical treatments need to be considered. Surgical treatments include peripheral techniques (such as cryosurgery, neurectomy, laser, acupuncture, thermocoagulation, injections of streptomycin, alcohol and phenol), Gasserian ganglion radiofrequency thermocoagulation, glycerol, balloon compression, Gamma knife and microvascular decompression. All the surgical treatments can result in nerve damage except microvascular decompression, which limits the application of these techniques. We faced with the challenge when some elder patients cannot tolerant surgical treatment.

In 2002, analgesic effect of BTX-A in TN was reported [14]. Some open studies [17] and a randomized controlled trial (RCT) [6] showed that BTX-A could significantly relieve pain in TN. The subcutaneously injection usually was adopted in many points[9,21],or muscle injection in region of the zygomatic arch [18] or in masseter [19]. But the dose has not been standardised. Based on our previous experience, the highest dose is 170U in a woman with the third branch injection, and the pain was completely relieved for 2 years [6,13]. In the present study, the VAS scale between 25U and 75U group were no significant difference from the first week to eighth week. However, the high dose led to facial weakness with high risk in our study.

Pain relief was observed in several minutes after injection [7], usually several days or as long as 20 days after injection [13]. In this study, the VAS scale was different between BTX-A and control group in the first week. The pain was relieved rapidly. The effect of BTX-A on TN usually lasted for several months [18,22]. Based on our previous experience, it may last for from 8 weeks to 2 years, but it was still unknown whether effect duration was associated with dose [6,13]. It was felt that the duration of pain relief sometimes was longer than the period of muscle weakness in our study. Because of this, it was considered that the antinociceptive effect of BTX-A may not associate with inhibiting the release of acetylcholine from the neuromuscular junction.

More patients in 25U and 75U group presented that they were “much improved” or “very much improved” than control in PGIC, but it was similar between 25U and 75U group at the endpoint. The effective rate was not significant different between 25U and 75U group, but they were different with control group. Complications induced by BTX-A were mild, including regional weakness, facial asymmetry during voluntary movement and edema in injection sites. Regional weakness and edema were transient and usually recovered within several weeks, and there were no systemic complications. Recent studies showed that BTX-A may reduce neurogenic inflammation by inhibiting the release of glutamate, substance P and calcitonin gene-related peptide (CGRP) in sensory terminal [23]. BTX-A may also reduce the release of substance P in dorsal root ganglia [24]. In other animal studies, BTX-A inhibits CGRP release in trigeminal sensory neurons of brainstem [25]. Diminishing peripheral sensory nerve chemical stimuli may contribute to decreased peripheral and central nervous system responsiveness [26]. More research is needed to explore the mechanism of action of BTX-A on pain in TN. BTX-A or its degradation product could achieve analgesic effect through indirect effects on spinal cord by reducing the transmission of the sympathetic nervous system or diminishing the inhibition of inhibitory interneuron renshaw cell [27]. Other Study of BTX-A treatment of migraine indicated that the analgesic effect of BTX-A not only associated with inhibiting the release of acetylcholine, but also associated with blocking the parasympathetic nervous system [28].

Taken together, our study suggested that BTX-A injection in TN was safe and efficient. It is a useful treatment for refractory TN. Lower dose (25U) and high dose (75U) were similar in efficacy in short-term. But we cannot claim that they were same in the long term. More studies are needed to clarify it in the following study.

### Research highlights

1. BTX-A injection in TN is safe and efficient.

2. BTX-A is a useful treatment for refractory TN.

3. Lower dose (25U) and high dose (75U) were similar in efficacy in short-term.

## Competing interest

The authors declare that they have no competing interest.

## Authors' contribution

Y-JL - Study concept and design. H-FZ - carried out the treatment and studies, drafted the manuscript Y-QM - acquisition of data. C-HH - participated in the sequence alignment, analysis and interpretation. DYC - revise the manuscript. N-CX, C-JW - study supervision. All authors read and approved the final manuscript.
